# Relationship Between Engagement with Life and AARC in Older Adults Over 80

**DOI:** 10.1093/geroni/igaf122.3073

**Published:** 2025-12-31

**Authors:** Natsue Shiraishi, Roman Kaspar, Takeshi Nakagawa, Yasuhiro Yamamoto, Ally Brothers, Takashi Horiuchi

**Affiliations:** Okayama University, Okayama, Okayama, Japan; Charlotte Fresenius Hochschule, Cologne, Nordrhein-Westfalen, Germany; The University of Osaka, Osaka, Osaka, Japan; Okayama University, Okayama, Okayama, Japan; Colorado State University, Fort Collins, Colorado, United States; Okayama University, Okayama, Okayama, Japan

## Abstract

The AARC theoretical model (Diehl et al., 2010) indicates the need to consider Engagement with Life. Kaspar et al. (2022) examined it in older adults aged 80 years and older, but their scope was limited. Therefore, this study examined the relationship between Engagement with Life and subjective aging (AARC). In this context, we investigated both the perceived importance and the actual frequency of activities. A cross-lagged model analysis was conducted using data from the German NRW80+ project, including data from 909 participants aged 80 and older over a two-year period. The results showed that higher importance of “creative activities” at Time1 predicted lower AARC-Losses at Time2 (β = -0.113). Additionally, higher AARC-Gains predicted higher importance of “solitary activities” for having peace and time for yourself (β = 0.137) and “mental activities” to deal with something in greater depth or to study a topic in more detail (β = 0.122). Higher frequency of “creative activities” predicted lower Losses (β = -0.132). Frequent participation in “mental activities” predicted higher Gains (β = 0.116). Higher Gains predicted higher frequency of “physical activity” (β = 0.093) and higher frequency of “mental activity” (β = 0.114). Frequent participation in “social activities” such as spending time with others like relatives or friends predicted lower Gains (β = -0.098). Higher frequency of “mental activities” (lifelong learning) predicted positive subjective aging, and the reverse was also observed. Higher importance and frequency of “creative activities” also predicted lower negative subjective aging.

